# Effect of *Moringa oleifera* Protein‐Coated Gold Nanoparticles as Naturally Derived Disruptors of Biofilms

**DOI:** 10.1155/ijbm/1381604

**Published:** 2026-02-11

**Authors:** Lakshmi Sudhir Menon, Narendranath Ghosh, Sumit Biswas

**Affiliations:** ^1^ ViStA Lab, Department of Biological Sciences, BITS Pilani KK Birla Goa Campus, Zuarinagar, Goa, 403726, India, bits-pilani.ac.in; ^2^ Department of Chemistry, BITS Pilani KK Birla Goa Campus, Zuarinagar, Goa, 403726, India, bits-pilani.ac.in

**Keywords:** bactericidal, biofilm, biofilm disruption, gold nanoparticles, leaf protein, *Moringa oleifera*

## Abstract

**Background:**

Microbes having affinity to metallic surfaces develop a highly structured and sessile colony known as biofilms which poses significant challenges to the domain of implant surgery. Biofilm‐mediated infections and the associated burden have prompted the search for multipronged approaches to tackle the problem. Gram‐positive pathogens, most notably *Staphylococcus aureus*, has been known to colonize human tracts and form biofilms on implants and prosthetic devices. Antibiotic tolerance, drug efflux, and recalcitrance to the host immune response have added to the existing predicament of the biofilm infections.

**Objective:**

The search for inexpensive but effective avenues for combating biofilms has led to the use of metal nanoparticles conjugated with plant‐derived proteins.

**Methods:**

In this study, a protein from *Moringa oleifera* leaf extract, p62, which has been previously identified to have antibiofilm properties, was conjugated with spherical gold nanoparticles (AuNPs) to target *S. aureus* biofilm formation.

**Results:**

The adsorption of p62 on the AuNPs was confirmed through microscopy, and the kinetics of binding was determined by plasmon resonance. The p62 coated AuNPs remained stable in solution and caused the successful disintegration of mature biofilms, more efficiently than the protein alone. The p62‐AuNPs were also found to disrupt the morphology of the cocci and cause cell death as evidenced from the live/dead cell imaging through confocal microscopy. The protein and the nanoparticle were not cytotoxic to C2C12 human myoblast cell lines, affirming their suitability to be used on implants.

## 1. Introduction

Biofilm‐mediated infections pose a major challenge to the medical fraternity causing increased morbidity and mortality, despite the availability of an array of antibiotics and antimicrobials at their disposal. Biofilms are resistant to antibiotics, and harbor increased antibiotic resistance due to their ability to cause drug efflux. The cytotoxicity of chemical therapeutic molecules and the current understanding of the molecular mechanisms of biofilm development have led to a restrategizing of treatments for biofilms‐associated infections [1, 2]. Among the strategies employed, a potent approach has been the use of metal nanoparticles conjugated to bioactives as antimicrobial agents.

Among biofilm‐mediated infections, *Staphylococcus aureus* has been concomitant with nosocomial infections on medical implants and prosthetic devices. Being a common dweller on the human skin, this Gram‐positive bacteria can switch from their planktonic to sessile form, establishing a slimy matrix of exopolysaccharides leading to the formation of a biofilm [3, 4]. Biofilms provide a favorable and defensive environment for further attachment and colonization on metallic (or even nonmetallic) surfaces of these indwelling devices, thus enabling resilience towards host immune response and systemic antibiotic treatment [1, 5].

Nanoparticle‐based medical applications have gained much momentum in recent times due to their heterogeneous surface properties, customizable shape and size, large surface‐to‐mass ratio, and most importantly biocompatibility. Metal and metal oxide nanoparticles (Au, Ag FeO_3_, TiO_2_, and ZnO) have exhibited promising results in bioimaging, gene and drug delivery, cancer treatment, therapeutics, and theranostics [6, 7]. They have been increasingly used to target pathogenic infections as a substitute for antibiotics and even for nanoparticle‐mediated biofilm disruption [8–10]. A wide variety of such delivery vehicles, viz. superparamagnetic ion oxides nanoparticles (SPIONs), hydroxyapatite aquasomes, and liposomes, have been explored for target‐specific and dual drug delivery (drug and bioactives) [11].

Nanoparticle drug delivery system (NDDS) provides a potential alternative to target multidrug resistance by rendering a way to encapsulate or bind drugs and antimicrobials to nanomaterials like solid‐core nanoparticles, lipids (Doxil, Paclitaxel), polymers etc., which can dodge the drug efflux mechanism [12, 13]. These systems have been explored a typical attachment with synthetic or biopolymers mainly polyethylene glycol (PEG), poly(lactic‐co‐glycolic acid (PLGA), polyethyleneimine (PEI), chitosan, dextran, and poly‐L‐lysine (PLL) that facilitates binding of proteins, enzymes, DNA, and drugs [14–16]. This property of NDDS provides solutions of improved design, and can be modified to suit a diverse range of therapeutic cargo, bioavailability, solubility, stealth effects, and low immunogenicity during application​ [13, 17, 18]. Moreover, the particle chemistry of metal nanoparticles allows effortless encapsulation and conjugate formation with any kind of biomolecules (enzymes, antibodies, growth factors, hormones, and toxins) leading to improved efficacy as drug moieties in the treatment of various physiological conditions [6, 10].

In search of novel antibiofilm agents, several classes of plant‐derived compounds—phenols, essential oils, alkaloids, terpenoids, and polypeptides/proteins—have been deployed, showing promising results with reduced toxicity and effective antimicrobial properties [2]. *Moringa oleifera* Lam. is a phanerogamous plant belonging to the Moringaceae family, commonly known as the horseradish or drumstick tree. It is native to Asia and is known for its ability to grow fast and for being an excellent remedy for malnutrition, being a very rich source of protein [2, 19]. Information on the uses of *M. oleifera* plant parts had been listed since centuries for use in both culinary practices and traditional medicine, forming the basis for current research and characterization of its components. All parts of this tree have been reported to have multiple benefits, especially the leaves which are a rich source of bioactives (minerals, vitamins, and phytochemicals) with curative potential for more than 300 diseases [19, 20]. Phytochemical extracts from different parts of *M. oleifera* have also been explored for antimicrobial, antioxidant, anticancer, antidiabetic, and antibiofilm properties [21]. However, literature has very limited resources available for proteins extracted from *M. oleifera* despite its rich protein content which has been claimed to be nine times than in yogurt [19].

In this study, we have extracted proteins from dried *M. oleifera* leaves which showed significant effects on static‐grown *S. aureus* biofilm cultures [22], both as a crude extract and, more effectively, when treated with the purified protein p62. Gold nanoparticles (AuNPs) were kinetically synthesized from HAuCl_4_, which were then coated with the purified moringa leaf protein p62. The adsorption of p62 on surface of AuNPs is designed by exploiting the polyvalent interactions offered by the protein side chains, which conciliates the need of any surface modification on the nanoparticle for interaction. The stability of the coated nanoparticles was confirmed through UV and IR spectrometry, along with the estimation of size and homogeneity. The presence of the coating was confirmed with scanning electron microscopy (SEM) and high‐resolution transmission electron microscopy (HR‐TEM), followed by selected area electron diffraction (SAED) of the p62‐coated AuNPs. Finally, the binding of the protein to the nanoparticles was verified from the surface plasmon resonance while using the protein as an analyte against immobilized AuNPs on a SA (streptavidin) chip. The p62‐coated AuNPs thus characterized showed effective disruption of *S. aureus* biofilms and could be promulgated as a potential antibiofilm agent that could be used in dressings or as coatings for surfaces of implant devices. Moreover, the components were fairly biocompatible when tested against human C2C12 cell lines, indicating their applicability for probable use on prosthetic devices.

## 2. Materials and Methods

### 2.1. Chemicals

Gold chloride HAuCl_4._3H_2_O (> 99%), phenylmethylsulphonyl fluoride (PMSF), ammonium sulfate NH_4_OH (> 99%), and HEPES (> 99%) were purchased from Sigma‐Aldrich. Trisodium citrate, potassium chloride KCl, dipotassium hydrogen phosphate K_2_HPO_4_, and monopotassium phosphate KH_2_PO_4_ were purchased from Merck. The growth media for bacterial culture used were LB from Himedia. The columns HK‐16, HiTrap Q‐HP anionic exchange, and resins Superdex 200 prep grade that were used for protein purification were purchased from GE Healthcare Life Sciences, Sweden. The solutions and buffers were strictly prepared in autoclaved Milli‐Q water throughout the experiment. All glassware was cleaned with chromic acid and dry autoclaved before use.

### 2.2. Synthesis of AuNPs

AuNPs were synthesized by employing the temperature‐controlled reduction of HAuCl_4_ with trisodium citrate in an aqueous medium followed by temperature‐controlled kinetic seeding process. Detailed methods for the synthesis of AuNPs are provided in the supporting documents (Section S1). The synthesized materials were subjected to dialysis using a SpectraPor membrane having a 2 kDa cutoff and analyzed with respect to particle charge, size, and size distribution using zeta potential analyzer (conducted at 25°C, at a detection angle of 173° backscatter), FE‐SEM, and DLS. The particles were further characterized with respect to their stability and composition using UV–Vis spectrophotometry, FTIR, EDS, HR‐TEM, and SAED. The details of the sample preparation and conditions have been listed in the supporting files (Section S2).

### 2.3. Extraction and Purification of p62 Protein

Mature *M. oleifera* leaves were hand‐picked from South Goa, India, and dried under shade for 2 weeks. Upon complete drying, the leaves were ground into a fine powder and used for extraction. The aqueous extract was obtained by dissolving 1 g of leaf powder in 6 mL (1:6 w/v) of extraction buffer (10 mM potassium phosphate, pH 7) with freshly prepared 10 mM protease inhibitor, PMSF. The mixture was then concentrated and subjected to chromatography to get the purified p62 protein as per the method devised previously [22].

### 2.4. Coating of p62 on AuNPs

The purified p62 was coated over the AuNPs with minor modifications to the method reported in Bastus et al. [23]. Briefly, 100 μL of a solution of 100 mM HEPES buffer (pH 7) was added to 500 μL of synthesized AuNPs (from a stock of 0.25 mM). Then, 100 μg of purified p62 (from a stock of concentration 0.5 mg/mL as determined by the Bradford method) was added to the AuNPs, and the final volume was adjusted to 1 mL with Milli‐Q water, followed by overnight incubation at room temperature.

The p62‐coated nanoparticles were diluted in water in the ratio of 1:1 and dropcast on a carbon‐coated copper 200 mesh (Formvar, Sigma) and visualized under TEM (JEOL‐1400 Plus) with an accelerating voltage of 120 kV. Bare nanoparticles were used as comparative controls to check for the formation of the protein corona on the coated ones.

To assign the elemental constitution of the AuNPs, p62‐coated nanoparticles were dropcast and dried on the SEM stubs covered with an aluminum wafer, and recorded using EDAX‐Ametek detector with FE‐SEM. The procedure was repeated for both the coated and the bare AuNPs, and a comparative assessment of the elements was undertaken.

### 2.5. Characterization of p62‐Coated AuNP

The structural characterization was also done for the p62‐coated AuNPs using DLS/Zeta, UV–visible spectroscopy, FTIR, SEM, and TEM (as detailed in the supporting files section S2). Intrinsic fluorescence of p62 was measured both in the presence and absence of AuNPs, with (*λ*_em_) 330 nm, after providing an excitation wavelength (*λ*_ex_) of 280 nm. The coated nanoparticles that were synthesized by mixing 100 μg of p62 at varying concentrations of AuNPs were further examined for protein quenching in a fluorescence spectrophotometer (JASCO, FP8200). The fluorescence intensity was used to plot the Stern–Volmer equation, stated in Equation (1), which helped in determining the type of quenching, binding constants (K), and type of interactions for all the interacting systems.

(1) f0f1=1+KsvQ,

where *f*_0_ and *f*_1_ are the fluorescence intensities in absence and presence of the quencher, respectively, Ksv is the Stern–Volmer quenching constant or association constant, and [Q] is the concentration of the quencher. Ksv was determined from the slope of the graphs plotted using Equation (1).

To understand the stability and interaction of the p62‐AuNPs, the spectral changes of the protein vis‐à‐vis the nanoparticle‐conjugated form were measured in a JASCO J‐1500‐150 CST spectrophotometer at 25°C. The spectrum obtained for both the protein and the p62‐AuNPs was analyzed using Spectra Manager 2 [24].

The molecular interactions between AuNPs and p62 were further studied by Surface Plasmon Resonance (Biacore T‐200) on Series S Sensor SA chip to understand the kinetics of interaction between the protein and AuNP. AuNPs were immobilized on the series SA (streptavidin) sensor chip using biotin‐NHS and PLL chemistry. Briefly, 100 μL of 1 mg/mL of biotin NHS was gradually mixed with 900 μL of 1 mg/mL of PLL (1:10) during continuous stirring for 2 h. The biotin–PLL complex was run through an Amicon concentrator (Millipore) to remove unbound biotin, followed by the collection of both filtrate and retentate separately. 200 μL of retentate was added to 800 μL of AuNPs (1:4) during continuous stirring for biotinylating the AuNPs. The final mixture was stirred for 2 h and centrifuged at 14, 000*g* for 15 min to remove unbound or nonbiotinylated AuNPs. The biotinylated AuNPs were then immobilized on the primed sensor chip as a ligand while the 100 μg of p62 was injected as an analyte.

Thereafter, kinetics studies were conducted with varying concentrations of the analyte (p62) at 0.25, 5, 10, 20, 80 nM. The SPR sensorgrams measured the SPR signals in response units (RUs) vs time, and the steady‐state binding model (1:1 binding) was ascertained using a fitting (Biacore Insight Software) to further determine the *K*_*D*_ value [25].

### 2.6. Culture and Treatment of *Staphylococcus aureus* Biofilms With p62‐AuNPs

*Staphylococcus aureus* (RN4220) cultures were inoculated in LB media and grown overnight in a shaking incubator at 37°C. For the growth of biofilm, the cultures were then transferred to static conditions for 5 days as per the previously mentioned protocol [22].

The biofilm cultures grown in static conditions were applied on a sterile coverslip and allowed to dry for 5 min. Coverslips with the biofilm thus prepared were incubated with purified p62 protein (at MIC [22]), bare AuNPs, and p62‐coated AuNPs along with a control (untreated biofilm) to understand the effect on the biofilm, if any. Similarly, cultures were incubated with p62, nanoparticles, and coated nanoparticles before static growth to check for the inhibitory action as well. The control and treated samples were stained with 2% glutaraldehyde and osmium tetroxide, followed by gradual dehydration using acetone gradients from 10% to 90%. For further dehydration, the samples were incubated in a critical point dryer (Leica) for 14 cycles for the removal of water.

To confirm the activity of the coated nanoparticles on the biofilm, these were also studied under the confocal microscope. Briefly, the biofilm culture was grown in confocal plates (VWR) under sterile conditions and treated with p62‐coated nanoparticles for one hour. The treated biofilm was stained with propidium iodide (PI) and SYTO9 from the Tracer Biofilm Live/Dead Assay kit (Invitrogen) in the same confocal plate. Treated and control cells (untreated biofilm) were stained with 10.02 mM of PI per mL of culture, and 60 mM of SYTO9 per mL for 30 min, and then washed with sterile water. The samples were observed in the confocal microscope (Olympus FV3000) with excitation at 488 nm and emission at 500–540 nm. The images obtained were analyzed using the ImageJ software suite (Olympus).

### 2.7. Determination of Cytotoxicity

C2C12 (CRL1772) myoblast cell lines were procured and used to check for cytotoxicity of the AuNPs. The cells were seeded into a 96 well plate at a density of 1 × 10^4^ cells/mL (as determined in a hemocytometer) and allowed to grow for 24 h in complete media (DMEM, 15% FBS, 1% antimycotic, antibiotic solution). 24 h postpassaging, the cells were treated with varying concentrations of p62 and AuNPs at concentrations 0.25, 0.20, 0.15, 0.10 and 0.05 mM and incubated for 24 and 48 h. After the treatment, the MTT reagent was added, and the assay was performed as per the manufacturer’s protocol (Roche, Cell Proliferation Kit I). Four hours after the addition of MTT reagent, the solubilization buffer was added and left for incubation overnight, following which the OD_570_ was recorded in a Multiskan GO plate reader (Thermo Scientific). Nanoparticles were similarly used to treat the myoblast cells to check for cytotoxicity.

## 3. Results and Discussion

### 3.1. Synthesis and Characterization of AuNPs

AuNPs were synthesized following the citrate capping method, and the physical characteristics such as the size, shape, and stability were determined. The diffusivity of AuNPs in solution yielded an average hydrodynamic size of 25 ± 5 nm with a polydispersity index (PDI) of 0.289 (Figure 1(a)) [26–28]. The citrate‐stabilized AuNPs have a net charge of −31.93 mV due to the multiple carboxylic groups associated with citrate (Figure 1(b)). All the measurements were performed in triplicate at different time intervals.

Figure 1(a) Hydrodynamic size measured for bare AuNPs and p62‐AuNPs using dynamic light scattering (DLS) and (b) measurement of electric potential of the samples using Zeta Sizer. (c, d) UV–visible spectra of synthesized AuNPs with a distinct peak at 540 nm at different hours and days. (e) A blue shift of *λ*_max_ in the spectra of p62‐AuNPs (524 nm) in comparison to bare NPs (528 nm).

**Figure:**
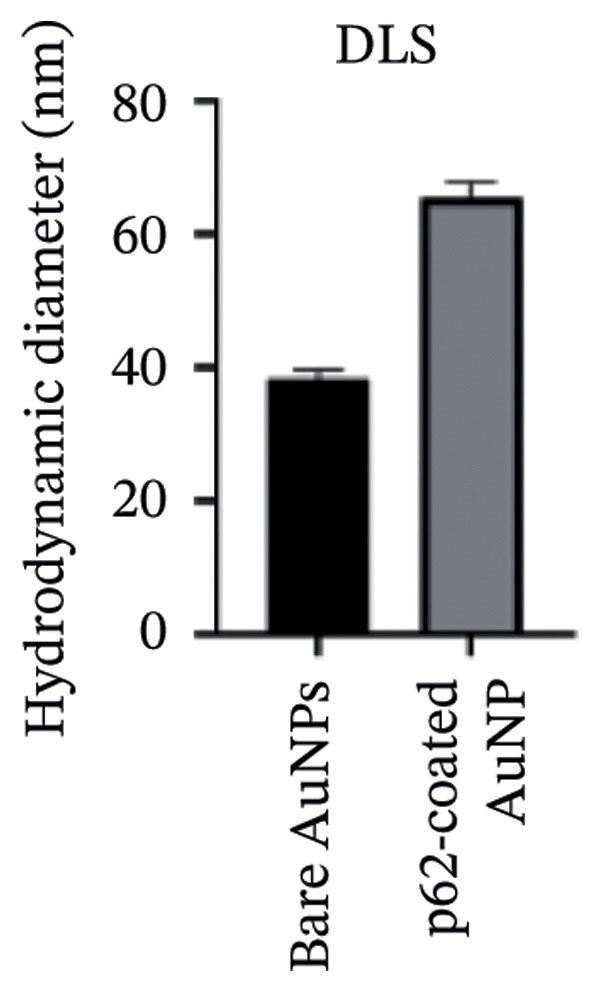
(a)

**Figure:**
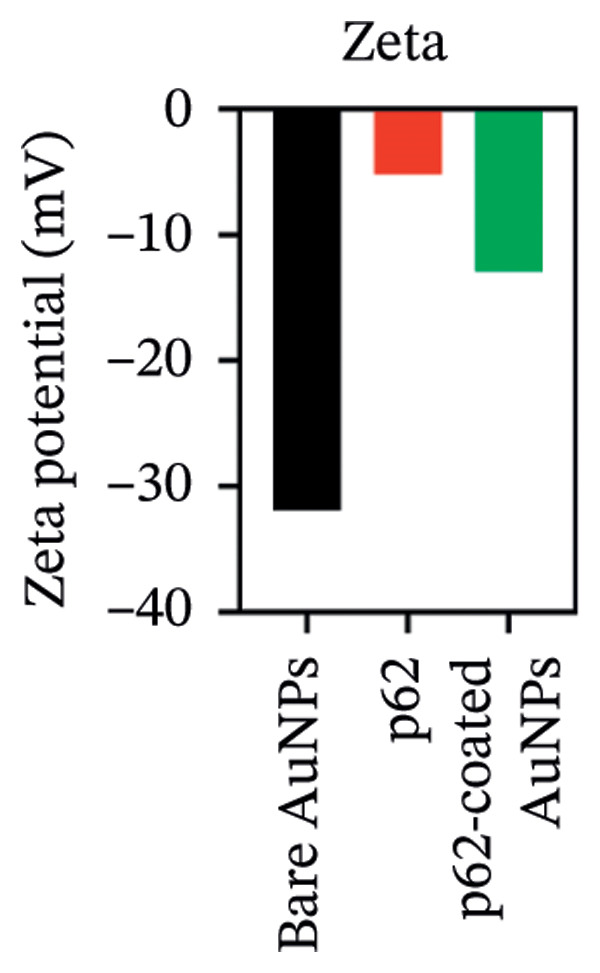
(b)

**Figure:**
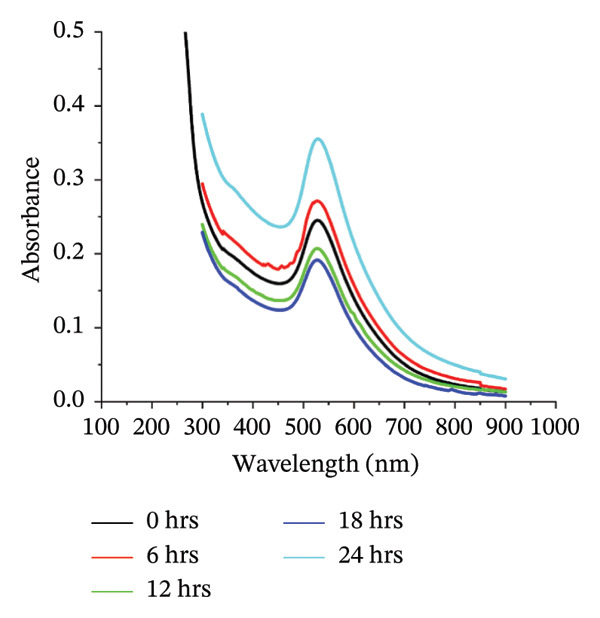
(c)

**Figure:**
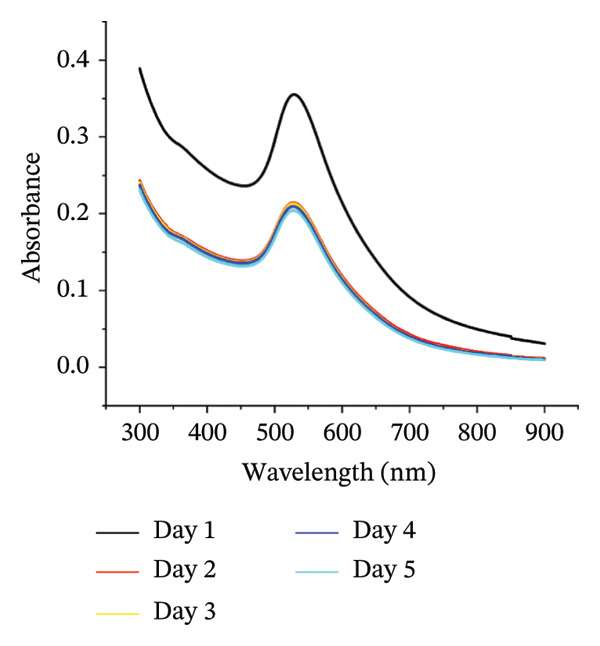
(d)

**Figure:**
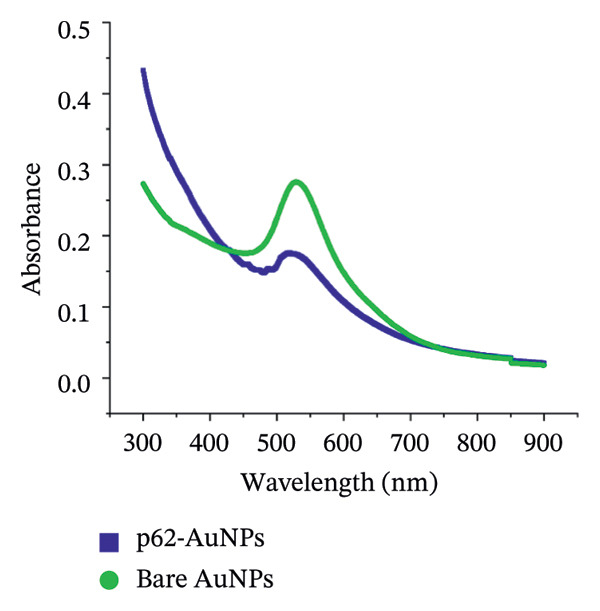
(e)

The UV–visible spectra of the wine‐red colored colloidal solution of the synthesized AuNPs show a plasmon band at 528 nm as shown in Figure 1(c), indicating an average size of ∼30 nm. The stability of this colloidal solution was checked from the spectra collected for several hours up to 5 days at periodic intervals. The *λ*_max_ of the plasmon band did not show any significant change with time, indicative of the stability of the AuNPs (Figure 1(d)). However, the particles form black agglomerates when kept for more than 30 days at room temperature, which though regain the wine‐red colored colloidal solution, if subjected to ultrasonication. The breakdown of the agglomerates with the characteristic plasmonic band at 528 nm was indicative of this transformation. For the synthesized AuNPs, the FTIR spectrum revealed resonance peaks at 3308 and 1636 cm^−1^ confirming the presence of the ‐OH and COO^−^ groups in the colloidal solution of AuNPs (Figure 2(a)).

Figure 2FTIR spectrum of (a) bare AuNPs and (b) p62‐AuNPs.

**Figure:**
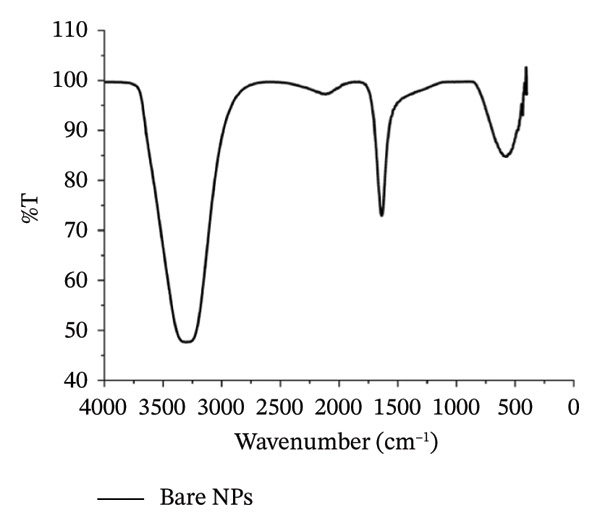
(a)

**Figure:**
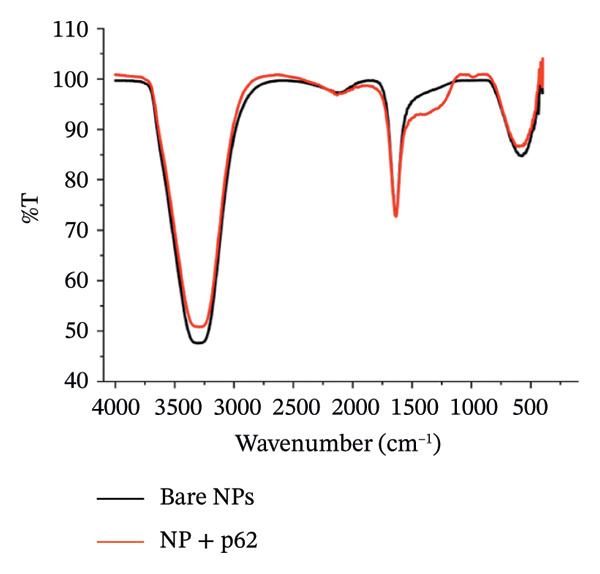
(b)

The FE‐SEM (Figures 3(a), 3(b)) and TEM (Figure 4(a)) images of AuNP showed spherical‐shaped particles, with an average size of 20.35 ± 2.06 nm and 19.02 ± 3.08 nm, respectively. The elemental analysis of the synthesized AuNPs with EDS yielded a presence of 7.15% Au (Figure 5(a)).

Figure 3SEM micrographs (a, b) show the synthesized AuNPs along with the corresponding size (in nm), while (c, d) show the p62‐coated AuNPs with size (nm). An average increase in size of 4 nm is observed in p62‐AuNPs as compared to bare AuNPs.

**Figure:**
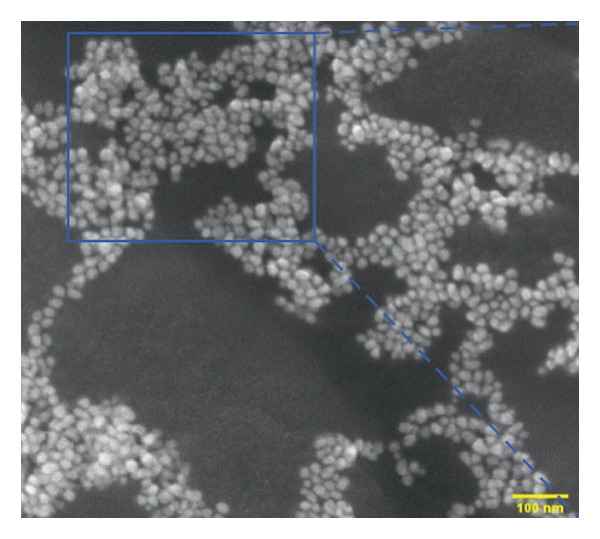
(a)

**Figure:**
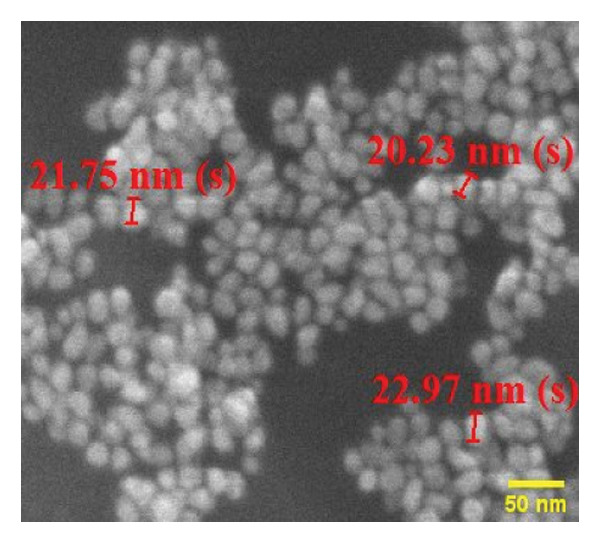
(b)

**Figure:**
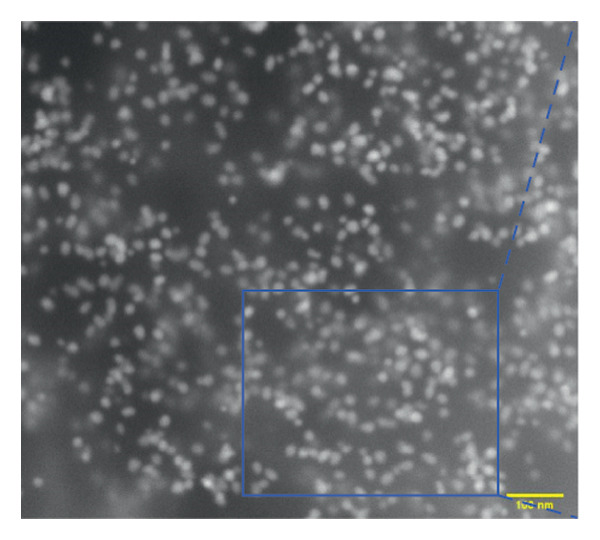
(c)

**Figure:**
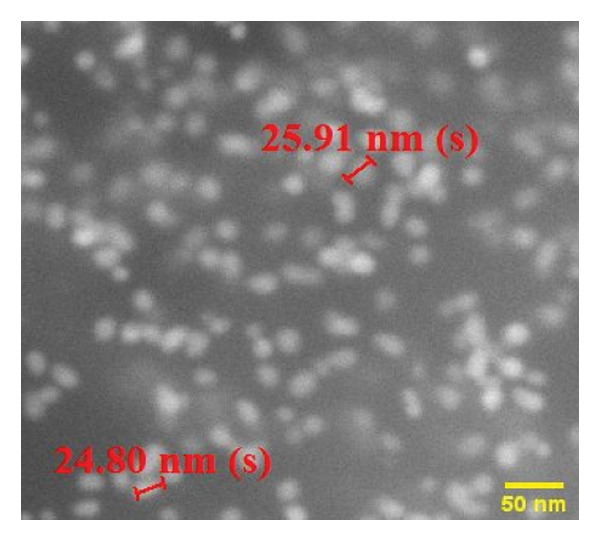
(d)

Figure 4(a, b) TEM & HR‐TEM micrograph of bare AuNPs at a magnification of 100 and 20 nm, respectively, where the average size measured for single particle was in the range of 20–25 nm, (c) HR‐TEM micrograph of bare AuNPs at magnifications of 2 nm, (d) length between the lattice fringes of bare AuNPs as observed and measured, (e) selected area electron diffraction pattern (SAED) of bare AuNPs showed diffraction spots associated with planes (1 1 1), (2 0 0), (2 2 0), (3 1 1), and (3 3 1), (f, g), p62‐AuNPs visualized under TEM and HR‐TEM at the magnification of 100 and 20 nm, the p62 protein corona over the AuNPs indicated by the blue arrows (inset), (h) HR‐TEM micrographs p62‐AuNPs at magnifications of 2 nm, (i) length between the lattice fringes of coated AuNPs as measured, and (j) SAED of coated AuNPs also showed diffraction spots associated with planes (1 1 1), (2 0 0), (2 2 0), and (3 3 1).

**Figure:**
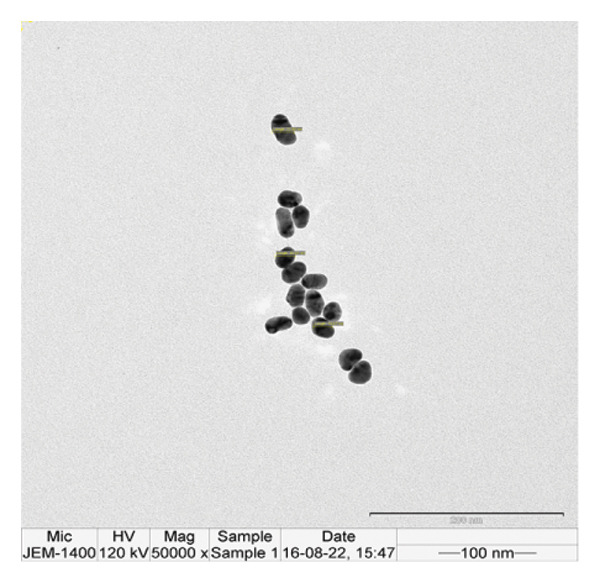
(a)

**Figure:**
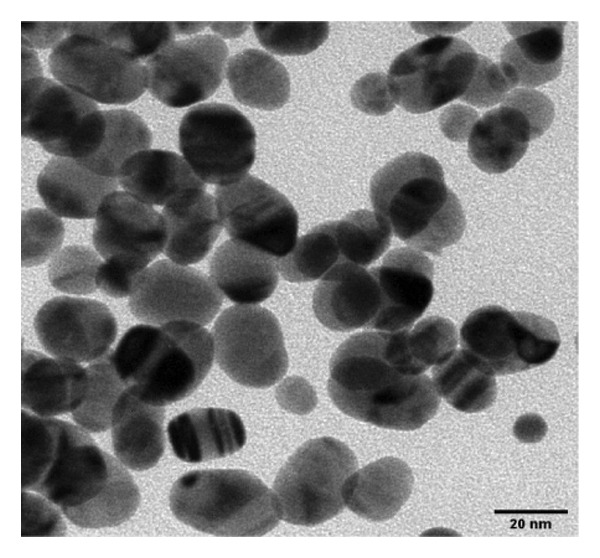
(b)

**Figure:**
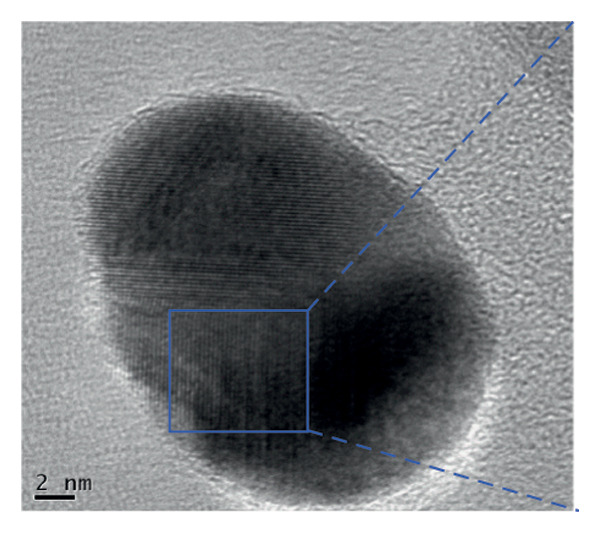
(c)

**Figure:**
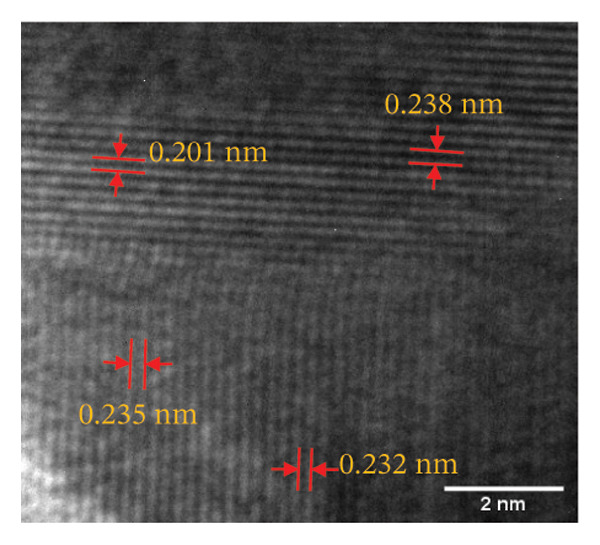
(d)

**Figure:**
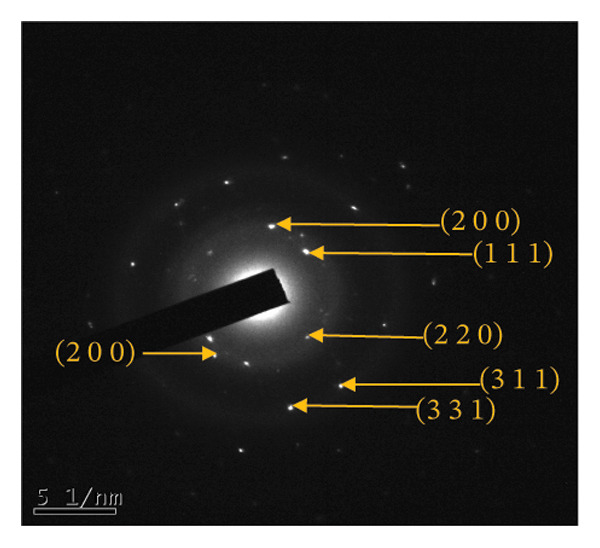
(e)

**Figure:**
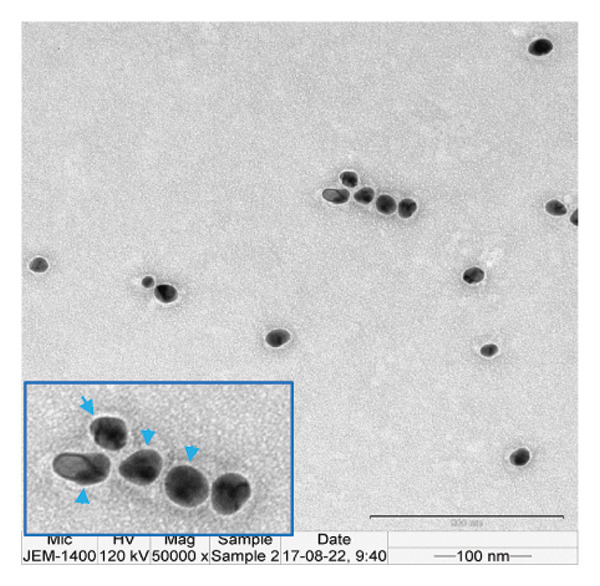
(f)

**Figure:**
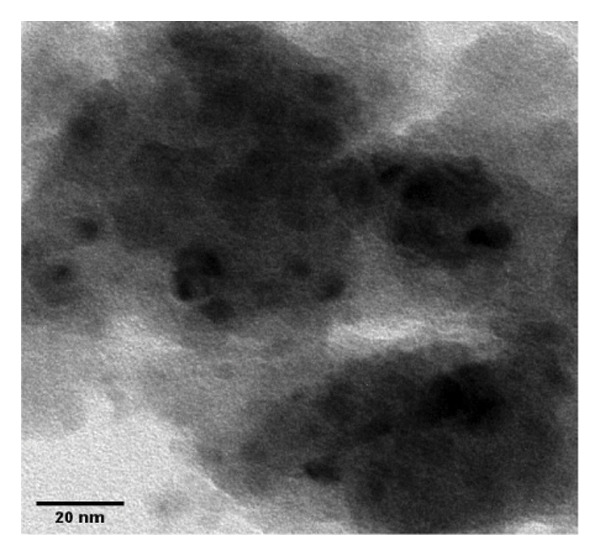
(g)

**Figure:**
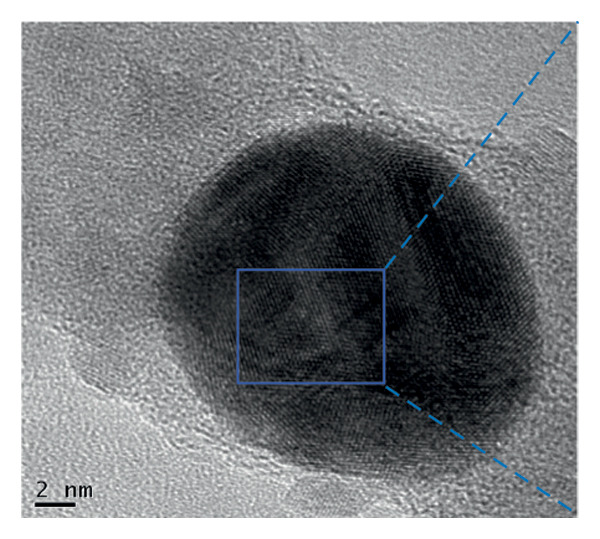
(h)

**Figure:**
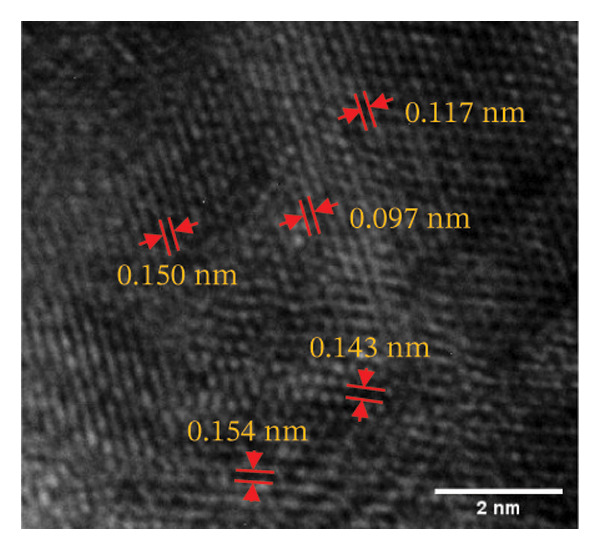
(i)

**Figure:**
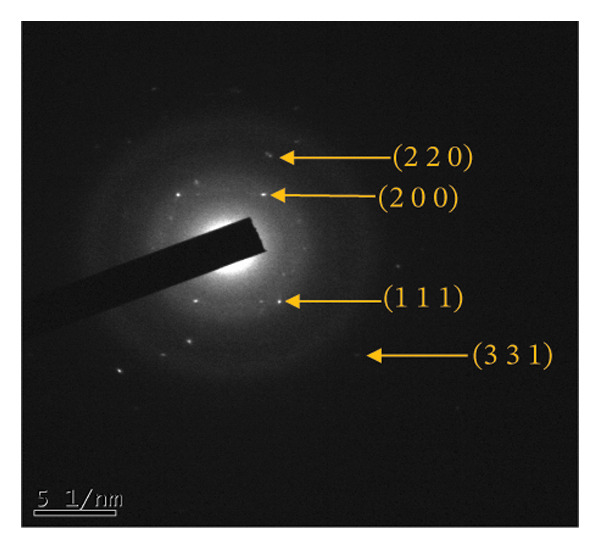
(j)

Figure 5EDS analysis of (a) bare AuNPs and (b) p62‐coated AuNPs.

**Figure:**
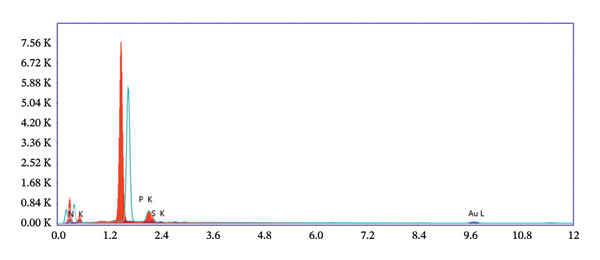
(a)

**Figure:**
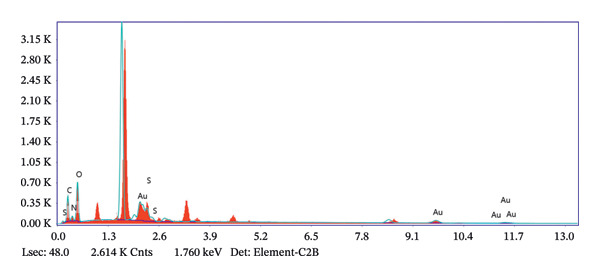
(b)

The HR‐TEM micrographs of the bare AuNPs showed lattice fringes (Figures 4(c) and 4(d)) having d‐values ∼2.35 and 2.04 Å associated with the planes (1 1 1) and (2 0 0), respectively (Figure 4(e)). The SAED patterns of the colloidal AuNPs revealed their crystalline structure (Figure 4(e)), and the diffraction spots at *d* = 2.35, 2.03, 1.23, and 1.45 Å for the (1 1 1), (2 0 0), (3 1 1), and (2 2 0) planes of gold were identified. The d‐values calculated for the colloidal AuNPs were corelated against the JCPDS data for AuNPs (PDF Card 00‐066‐0091).

### 3.2. Extraction, Purification, and Coating of p62 on AuNPs

P62 was isolated from the aqueous crude extracts of *M. oleifera* leaves and purified to homogeneity using size‐exclusion and anionic exchange chromatography techniques as shown in Figure S3 (supporting file). P62 was coated over the AuNPs with HEPES in the ratio of 5:1:1:3 (AuNPs: HEPES: water: P62). HEPES was added to maintain the pH and avoid precipitation or aggregation of AuNPs on interaction with p62. Also, as the protein and the AuNPs are both negatively charged, a salt was required to reduce charge‐based repulsion and facilitate the adsorption of protein over the nanoparticles, similar to the process followed in Wu et al. ​ [29]. Proteins interact with the AuNPs through many polyvalent interactions, mainly mediated by the thiol group. In contrast to the bare AuNPs, the p62‐coated samples were surrounded by a shiny halo/corona (Figures 4(f) and 4(g)) which indicates the interaction of the protein with the nanoparticle. The EDS analysis too confirmed the presence of around 6.38% of S in p62‐AuNP (untraced in the bare AuNPs), which could have been contributed only from the protein. The significant presence of sulfur supports our hypothesis of the thiol group of sulfur‐containing amino acids binding to the AuNPs (Figure 5(b)). In addition, the p62‐AuNPs also showed the presence of 16.72% N in the elemental analysis suggesting the presence of elements from the protein moiety (Figure 5(b)).

### 3.3. Interaction of AuNPs With p62 Protein

The zeta potential of the p62‐AuNPs was found to drop to −12.92 mV, indicative of interaction between the protein and the nanoparticles. The average hydrodynamic diameter (size distribution) of p62‐AuNPs, meanwhile, was calculated at 60 ± 7 nm with a 0.21 PDI after the absorption of the protein on the nanoparticles (Figure 1(a)). This value was supposedly larger than the sizes calculated from the SEM and TEM images. The variation in size between the TEM and the DLS can be attributed to the fact that TEM measurements are done on the basis of the smallest entities exposed to the beam. On the other hand, DLS is an intensity‐based estimation which is calculated on the basis of the larger particle sizes and the cumulative analysis of the hydration sphere diameter over several nanoparticles in solution [26]. The presence of the thin halo, around the AuNPs in the TEM micrographs, in the form of a protein corona also confirms the adsorption of p62 on the AuNPs (Figures 4(f) and 4(g)).

The UV–visible spectra for the p62‐AuNPs, when compared to that of the bare AuNPs, showed a blue shift with a plasmon band at 524 nm (Figure 1(e)). This shift towards shorter wavelengths is probably due to the rearrangement of the functional group of the AuNPs and the p62 [30]. However, the p62‐AuNPs maintained their stability over a period of time identical to the bare AuNPs.

The FTIR spectrum for the p62‐AuNPs showed a distinct shouldering between 1600 and 750 cm^−1^ which was not observed for the bare AuNPs (Figure 2(b)). These bands reveal scissoring vibrations of ‐CH‐ from the CH_3_N ^+^ group (1508, 1466, and 1406 cm^−1^), and less intense peaks of amide III vibrations were also observed from 1200 to 1400 cm^−1^. The presence of these spectral peaks pointed to the successful conjugation of p62 on AuNPs [31].

The changes in the emission spectrum of the chromophores (tryptophan, tyrosine, and phenylalanine residues) in proteins upon binding nanoparticles can provide a convenient method for understanding binding and conformation changes upon association with NPs [32–36]. Compounded with the fact that gold efficiently quenches the emission of these chromophores [37, 38], measurements of fluorescence quenching can elucidate the relative accessibility of gold NPs to protein chromophore groups. The fluorescence spectra of p62 showed a gradual reduction in intensity with the addition of AuNPs (Figure 6(a)). The protein concentration was maintained at 0.1 mg/mL, while the concentration of AuNPs increased from 0.05 to 0.2 mM. The sequence of the p62 determined earlier [22] showed the presence of significant amount of tyrosines and also other chromophores. The decrease in the intensity with increase in the AuNP concentration is indicative of the progressive increase in fluorescence quenching as it has been plotted from the Stern–Volmer equation (Figure 6(b)). Since the chromophores on the surface of the protein are in proximity to the AuNPs, and it is also these chromophores which were responsible for the fluorescence intensity, effective quenching is seen. The quenching of fluorescence was also associated with a substantial blue shift in the *λ*_max_, indicative of a shift of the dielectric properties of the medium, or reduction in the polarity of the medium, and corroborated also from the reduced zeta potential values of p62‐AuNPs [30, 39–41].

Figure 6(a) Fluorescence spectra of AuNPs with p62 in varying concentrations, (b) Stern–Volmer plot for deducing the dissociation constant *k*_*d*_ value for p62‐AuNP interactions, (c) circular dichroism spectra of p62 (blue) and p62‐AuNPs (green), (d) strategy for binding interaction on the SA chip: biotinylated (purple) gold nanoparticles (yellow spheres) were immobilized onto the SA chip via streptavidin (olive spheres), and the protein (blue strands) was used as an analyte, (e) association and dissociation of p62 at different concentrations when injected to the immobilized AuNPs on SA chip and analyzed in Biacore, (f) dose–response curve for interaction of p62 with AuNPs. The curve fitting for dose response plot was carried out using nonlinear regression for one site‐specific binding.

**Figure:**
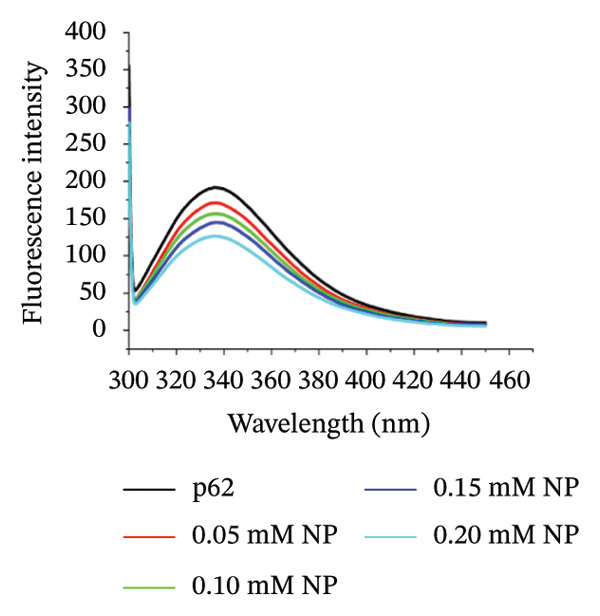
(a)

**Figure:**
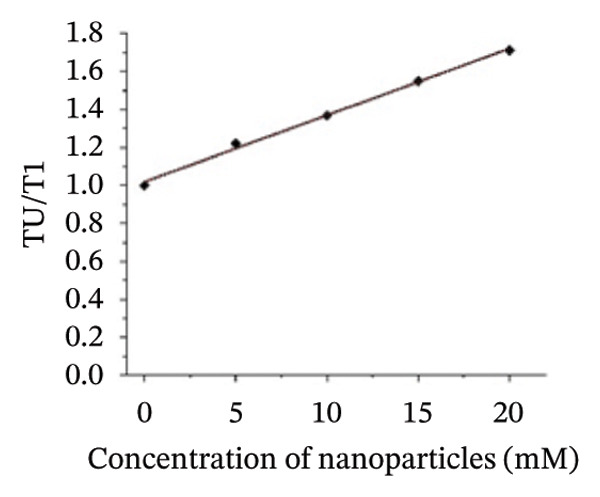
(b)

**Figure:**
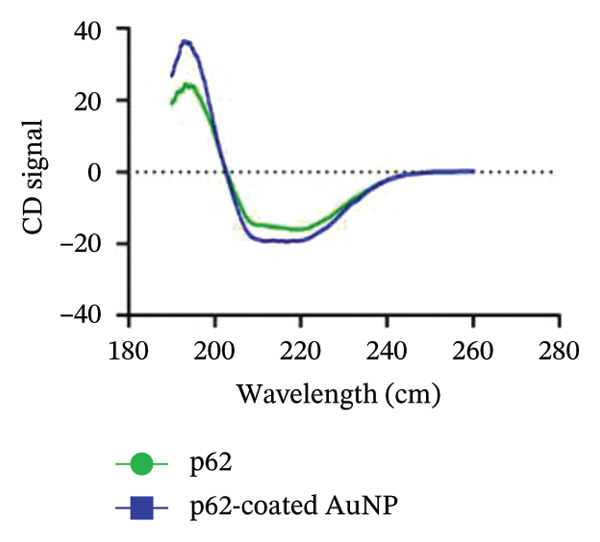
(c)

**Figure:**
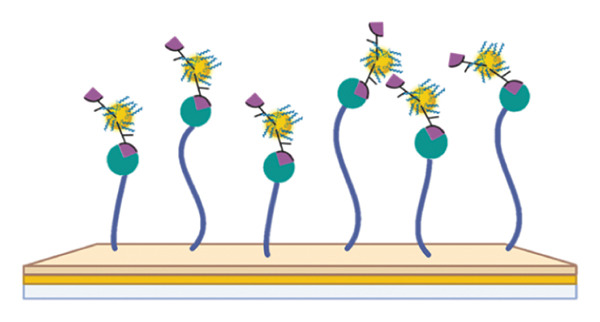
(d)

**Figure:**
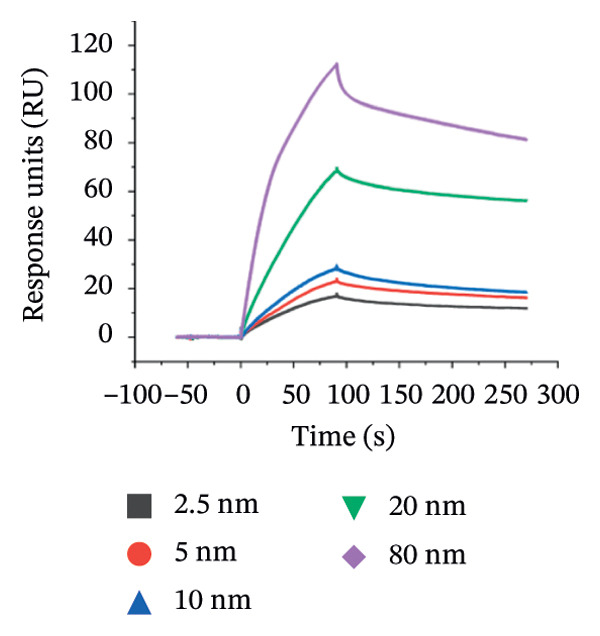
(e)

**Figure:**
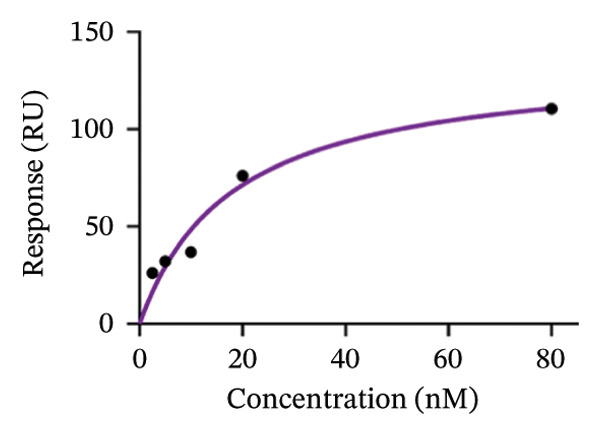
(f)

The CD spectrum for p62 (Figure 6(c)) showed an equivalent composition of alpha helices and beta sheets in the native form. The spectrum showed very little deviation for the protein coated nanoparticles, with respect to both the intensity and the secondary structure composition. This is indicative of the adsorbance of p62 on the AuNPs as well as the stability of the protein in the conjugated form.

From the diffraction spots observed in the SAED pattern of p62‐AuNPs, the obtained *d* values were 2.45, 2.08, 1.49, and 0.94 Å which are attributed to (1 1 1), (2 0 0), (2 2 0), and (3 3 1) planes of Au, respectively (Figure 4(j)). This indicated that the coating of p62 did not affect the crystalline structure of the AuNPs [42].

The interaction between the nanoparticles and p62 was studied using surface plasmon resonance (Biacore T‐200) at 25°C. The conventional EDC and NHS chemistry was avoided here. This is because the isoelectric point of p62 was quite close to that of the dextran (pI‐3.5) coating on the sensor chip. Hence, the experiment was designed to use the AuNPs as the ligand, while p62 was used as the analyte. Therefore, the AuNPs were immobilized on the SA chip with a final concentration of 0.2 mM in a two‐step process. Briefly, the PLL and biotin‐NHS were incubated together to form a PLL–biotin complex through the ‐NH_2_ group of lysine binding to the ‐COOH of biotin. The biotin–PLL was incubated with AuNPs further on (with gentle shaking) for a period of 3 h. This was then centrifuged in an Amicon column to collect only the retentate. These biotinylated AuNPs were then successfully immobilized on the SA chip on the basis of the avidin–biotin chemistry. The retentate was washed thoroughly with multiple washes before loading on to the chip, to enable the successful immobilization of PLL–biotin–AuNPs.

The biotinylated AuNPs were successfully captured on to the SA chip with a response of 1333.2 RU. Further, the p62 was injected as the analyte at varying concentrations of 2.5, 5, 10, 20, and 80 nM to explore the binding kinetics of the protein to the AuNPs. The sensorgrams (Figure 6(d)) demonstrated a high‐affinity interaction between the AuNPs and p62, exhibiting a concentration‐dependent binding, over an association time of 90 s. The association (1.711 × 10^6^) and dissociation (2.0307 × 10^−3^) rate constants of the binding reactions were arrived using the Langmuir global fitting model, to yield the calculated *K*_*d*_ value of 1.18667E − 09. The curve was fitted as per steady state model 1:1 binding to the theoretical *R*_max_ value of 104.5997629 (Figure 6(e)). The equation for the curve was not valid below concentrations of 2.5 nM, which were anyway too minimal to be effective. The binding curve though plateaued beyond 40 nM, which indicated the saturation of the binding sites and the absence of nonspecific interactions in the reaction complex.

### 3.4. *Staphylococcus aureus* Biofilms Growth and Treatment

*S. aureus* biofilms were grown for 5 days in static surfaces, and the mature biofilms were transferred onto coverslips. The coverslips were treated with AuNPs, the protein p62, and p62‐coated AuNPs. Compared to the untreated control, where the biofilm remains intact with an EPS matrix, there is a visible reduction of the biofilm integrity in the p62‐treated and p62‐AuNP treated samples (7 c and d). The effect is more pronounced for the p62‐AuNP samples where the severing of the biofilm is seen associated with disruption of the EPS matrix as well (Figure 7(d)). There is also an observed stress on the surface of the cocci which was evident after the treatment with p62 and p62‐coated NPs. In the case of the p62‐AuNP‐treated samples especially, there were visible protrusions from the cocci (Figure 7(e)) which were probably a precursor to the lysis of the cocci (as understood from the live/dead imaging results described further). Importantly, the biofilm samples treated with bare AuNPs did not show any disruption as such, but showed a thorough deposition of the nanoparticles on the biofilm‐embedded cocci, which were healthy in morphology (Figure 7(b)).

Figure 7SEM micrographs with (a) the mature biofilms in control, (b) effect of biofilm when treated only with bare AuNPs, (c) effects on the biofilm and coccal morphology when treated with only p62 protein (d, e) clear dispersion of the biofilm layer as well as loss of coccal morphology of individual cells when treated with p62‐AuNPs. Confocal microscopy using live/dead cell imaging. (f) *S. aureus* biofilms (control) appear green in color due to the absorption of SYTO‐9 (green fluorescence), while (g) p62‐treated cells show emission of red fluorescence (PI), whereas (h) cells treated with p62‐AuNPs show an intense emission of red fluorescence. The p62‐AuNPs cause cell death along with biofilm disruption, (i) the comparison between measurement of dead cells for p62 and p62‐AuNPs‐treated cell vs untreated control yielded a statistically significant difference (*p* < 0.05) for *N* = 3 sample size, and (j, k) the viability of C2C12 cells (myoblasts) on dose‐dependent treatment with both p62 and AuNPs.

**Figure:**
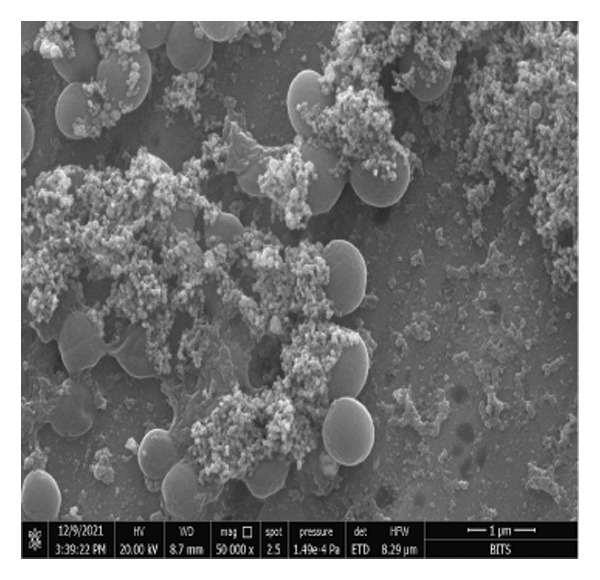
(a)

**Figure:**
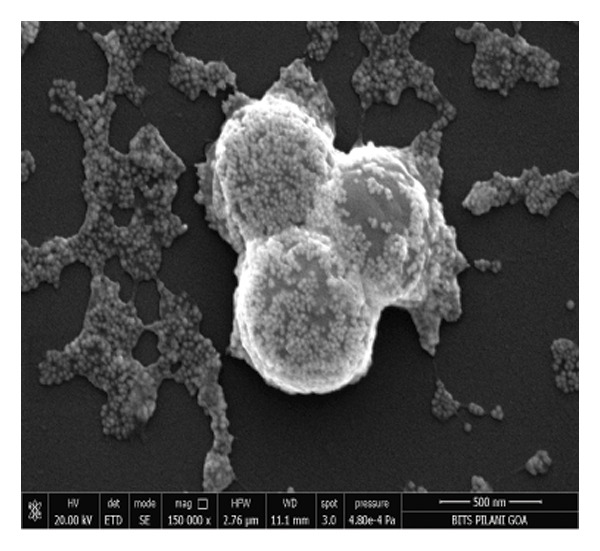
(b)

**Figure:**
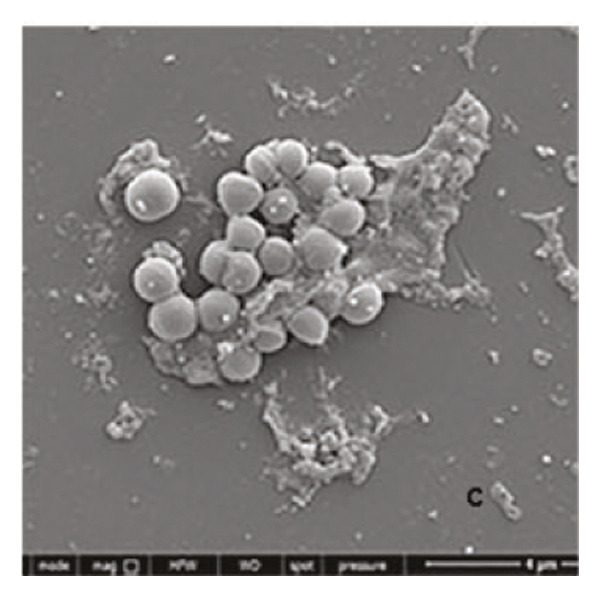
(c)

**Figure:**
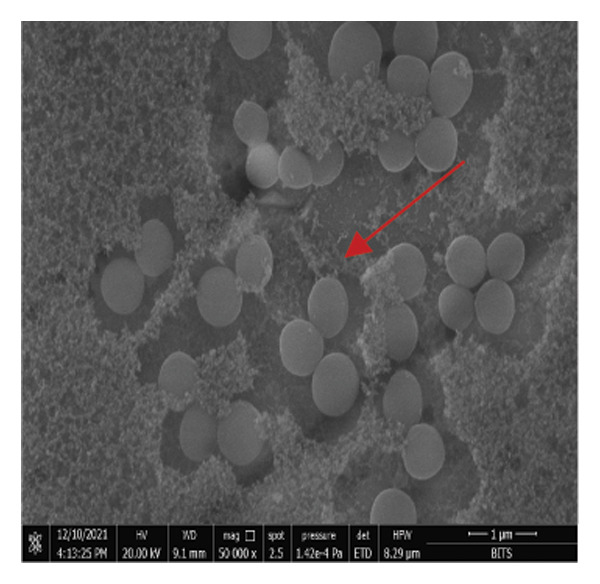
(d)

**Figure:**
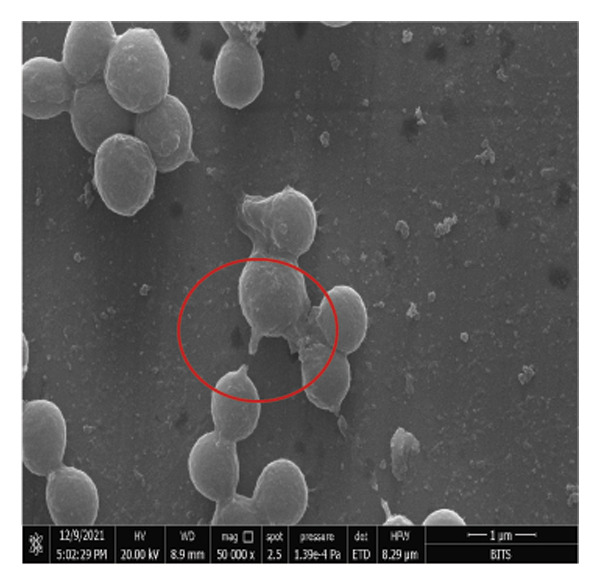
(e)

**Figure:**
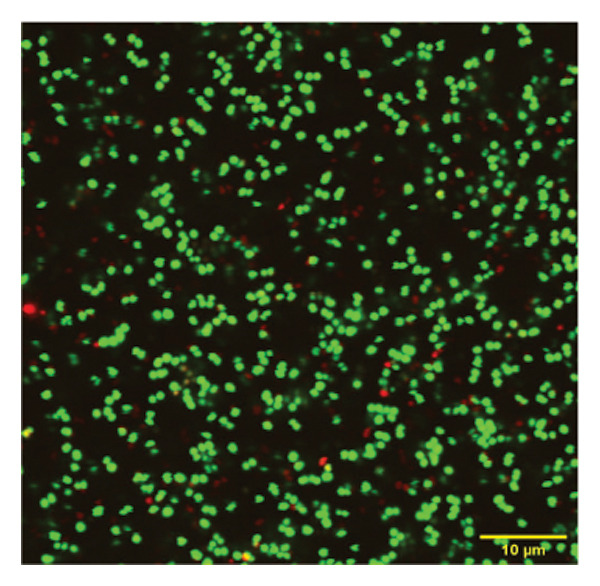
(f)

**Figure:**
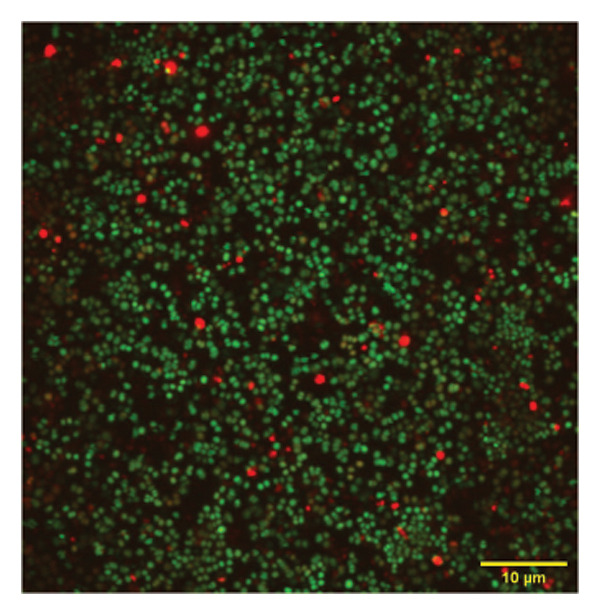
(g)

**Figure:**
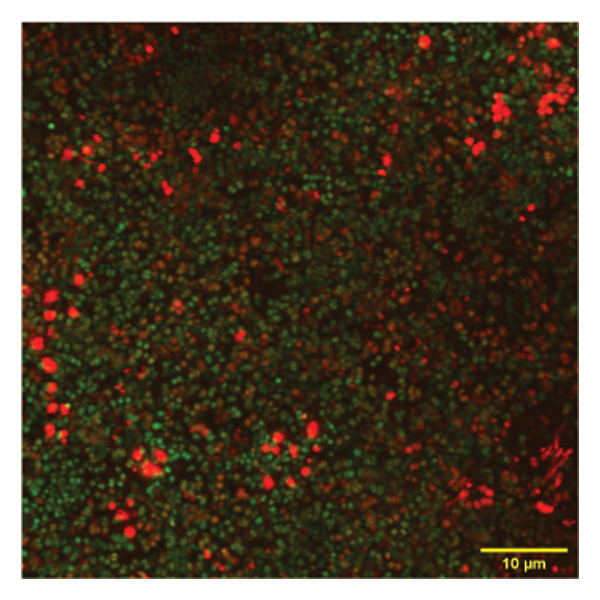
(h)

**Figure:**
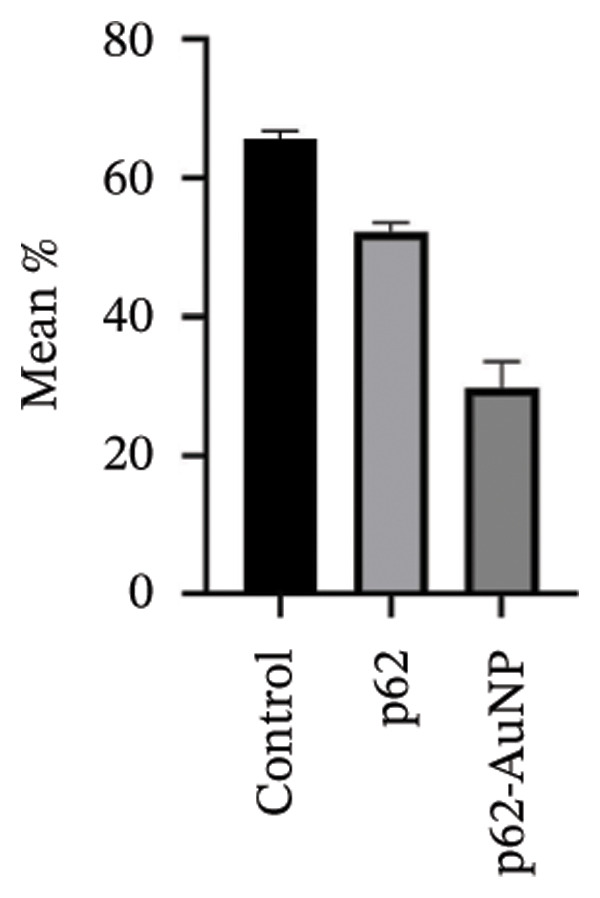
(i)

**Figure:**
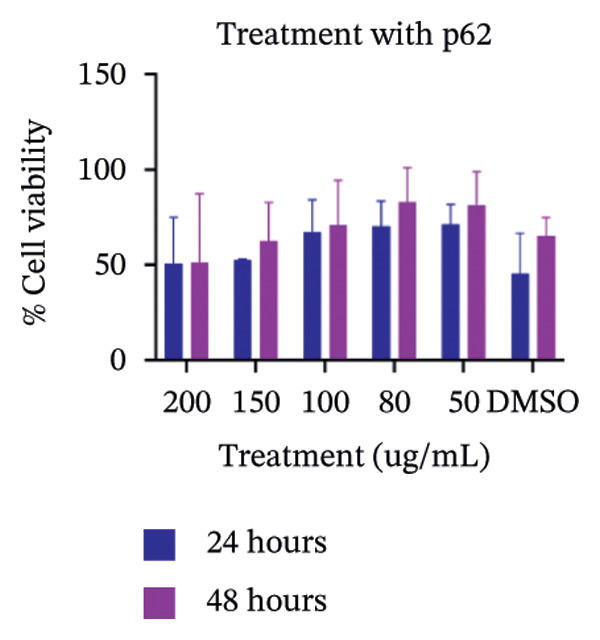
(j)

**Figure:**
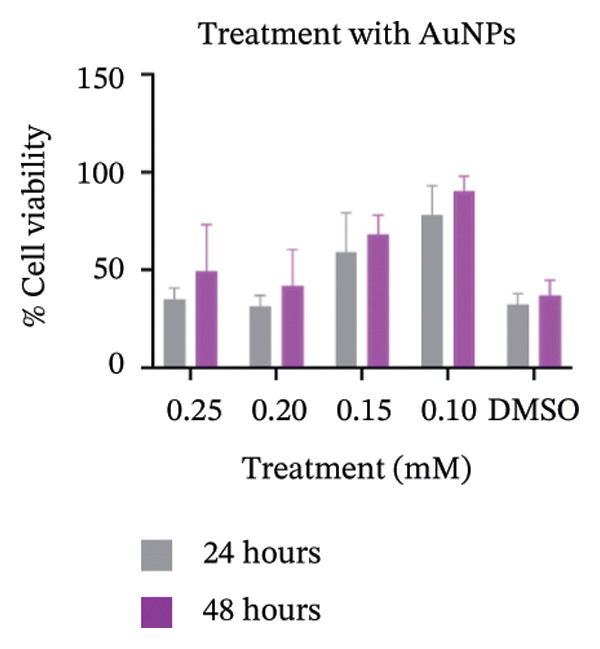
(k)

For further investigation of the antimicrobial potential of the coated nanoparticles, the treated biofilms were observed at intervals, keeping the untreated biofilms as control. As depicted in Figures 7(f), 7(g), and 7(h), the results were similar to the SEM observations. The SYTO9 of the live/dead viability kit stains all cocci, imparting a green fluorescence, whereas PI cannot penetrate those cocci where the membrane is intact. Thus, the predominantly green fluorescence observed in the untreated biofilm is indicative of the presence of mostly live and viable cells in the sample (Figure 7(f)). On the other hand, cocci whose membranes have been compromised allow the entry of PI, and hence, the red fluorescence dominates when the cells have ruptured membranes even though both the dyes are used in conjunction, a property observed in the biofilm samples treated with p62 (Figure 7(g)). The membrane rupture and eventual cell death is even more pronounced when the p62‐AuNPs are added to the biofilm samples, resulting in a stronger PI fluorescence (Figure 7(h)). The results have also been corroborated from crystal violet assays (result not shown). The fluorescence intensity of the live cells (green fluorescence) was measured for quantification of coccal viability after treatment with p62 and p62‐AuNPs and was analyzed using ANOVA which yielded a *p* value < 0.05, suggesting that along with biofilm disintegration, there is also significant decrease in the number of viable coccal cells when treated with p62 and p62‐AuNPs.

### 3.5. Cytotoxicity

Cytotoxicity analysis was performed by using an MTT assay on C2C12 cell lines. Myoblast cell lines were used since the destination of the implants would be primarily the skeletal muscles which would be in close conjunction to the coated nanoparticles *in vivo*. The cell lines were treated both with p62 and nanoparticles. The highest concentration used for p62 was 150 μg which showed less than 50% cell viability for the 24 h treatment and 54% cell viability in 48 h. 100 μg of p62 (the minimum inhibition concentration) showed around 70% cell viability, whereas the viability did not improve significantly when 80 or 50 μg of the protein was applied. When C2C12 cell lines were treated with nanoparticles at various concentrations, 0.1 mM showed close to 80% cell viability after 24 h, whereas viability was 90% after 48 h. Increase in the concentration to 0.2 mM had adverse effects on cell viability, with the percentage of viable cells dropping to 31%–40% over both 24 and 48 h periods. The concentration of colloidal AuNPs selected for our study was approximately 0.125 mM, at which the cell viability can be safely concluded to be within reasonable limits.

Overall, the p62 protein which has been reported as a potent inhibitor of biofilms [22] has been coated on AuNPs synthesized by the citrate capping method. The synthesized nanoparticles were stable at room temperature and maintained all characteristic features as observed from their UV–visible or FTIR spectra. The stability of adsorption of the protein on the AuNPs has been studied and validated through multiple processes as this is a very important aspect for further effectiveness of the conjugated nanoparticles. The increase in size as observed from the SEM images or the hydrodynamic diameter has been validated with the presence of a protein corona on the AuNPs as visualized in HR‐TEM. EDS analysis of the coated nanoparticles also confirmed the presence of amino acids in the conjugates. The binding of the protein to the nanoparticles has also been observed through the fluorescence studies and more conclusively from the SPR kinetics. The p62‐AuNPs thus characterized were used to treat robust biofilms formed by *S. aureus*. Effective disruption of biofilms was seen in the treated samples when assessed through the traditional CV assay [22] or when visualized in SEM and confocal microscopy. These results support the efficacy of p62‐AuNPs as a novel biomaterial which can effectively disrupt biofilm formation and growth on solid substrates.

## 4. Conclusion

Biofilm formation at the sites of infection and at implants has been a threat to the medical fraternity owing to the associated costs and the burden of repeat surgery. The process of microbial colonization in most biofilms begins with Gram‐positive bacteria, especially *S. aureus*. Thus, the control of bacterial infection at the site of initiation of biofilm‐formation acquires utmost importance. Since it is difficult to address the initiation of growth in a system embedded in living tissues, the implant or prosthetic device should be enabled with resistant material that would inhibit the process of biofilm formation. To address this, a protein‐coated nanoparticle‐based coating material has been advocated in this work. The p62‐AuNP, with the p62 protein adsorbed on AuNPs in the form of a solid corona, has been seen to effectively inhibit biofilm growth *in vitro*. The p62‐coated nanoparticle remains stable, maintains its conformation during coating, and could inhibit as well as disrupt staphylococcal biofilms as seen both from SEM images as well as crystal violet measurements. Not only that, p62‐AuNPs render effective bacterial killing as seen from the live/dead imaging in confocal microscopy. Being reasonably biocompatible, this novel material obtained from relatively inexpensive plant source can be an effective means of countering the threat of biofilm growth on implants, when used as a coating for prosthetic devices.

## Conflicts of Interest

The authors declare no conflicts of interest.

## Funding

This study received funding from ICMR (OMI/4/2019/ECD‐I) for the scholarship to L.S.M. for the duration of some part of the work.

## Supporting Information

Section S1: The methodology used for synthesis of AuNPs, with exact concentration of substrate utilized and the conditions used are mentioned.

Section S2: The details of the sample preparation and conditions used for each of the analytical methods mentioned are explained in detail for both bare AuNPs and p62‐coated AuNPs.

Figure S3: Gel profile and elution profile for purification of p62 from *Moringa oleifera* leaf extracts after subsequent chromatography.

## Supporting information


**Supporting Information** Additional supporting information can be found online in the Supporting Information section.

## Data Availability

Data are available on request from the authors.
