# Supplementary material for: Effect of Moringa oleifera Protein‐Coated Gold Nanoparticles as Naturally Derived Disruptors of Biofilms
Source: Int J Biomater. 2026 Feb 11;2026:1381604. doi: 10.1155/ijbm/1381604 (PMC12892876; doi:10.1155/ijbm/1381604)
Supplement: Supplementary file 1 — Supporting Information Additional supporting information can be found online in the Supporting Information section. [file IJBM-2026-1381604-s001.docx]

**SUPPLEMENTARY DATA**

**S1. Synthesis of AuNPs.**

Gold nanoparticles (AuNPs) were synthesized by temperature-controlled kinetic seeding reduction of HAuCl_4_ as reported in Bastus et al (Bastús et al., 2011). Briefly, a solution of 0.25mM HAuCl_4_ was heated to the temperature of 100^o^ C in a two-necked round bottom flask. Under constant stirring at 770 rpm in a temperature-controlled oil bath, 15 ml of pre-heated (up to 50^0^ C) solution of 0.51mM sodium citrate dihydrate was seeded to 450 mL of boiling gold chloride solution. The reaction was allowed to progress for 15 minutes with steady stirring and boiling, after which the solution was allowed to cool at room temperature with continued stirring. The colour of the solution turned from golden yellow to grey and finally to wine red at the end of 15 minutes.

**S2. Characterization of AuNPs and p62-coated AuNPs.**

***Stability of Nanoparticles*** The spectral scans of the resulting gold nanoparticles was obtained from a UV-Visible spectrophotometer (Jasco V-570 UV/Vis/NIR Spectrophotometer) spanning a wavelength of 300-800 nm from 0 to 24 hours and for 7 days at 6-hour intervals and 24-hour intervals respectively.

***Size distribution and net charge.*** The size distribution, polydispersity index and net charge of the synthesized AuNPs were acquired with the help of Particle Analyzer (NanoPlus, Particulate Systems). The analysis was performed at 25℃.

***FTIR characterization*** For functional elucidation of gold nanoparticles, the AuNPs were analysed in IR (Perkin Elmer Spectrum IR Version 10.6.1)

***Visualization of AuNPs through Electron Microscopy*** Gold nanoparticles were dissolved in Milli-Q water in a ratio of 1:1 and dried overnight on an aluminium wafer. The dried AuNP sample was sputter coated in Leica EM ACE 200 with a 3nm thick layer of gold-palladium and then visualized under FE-SEM Quanta FEG 250 at a magnification of 150000-300000X. The size of the particle was measured using SEM-licensed software and co-related with the DLS data.

**S3. Purification of p62 from *Moringa oleifera* leaf extracts after subsequent chromatography.**

**
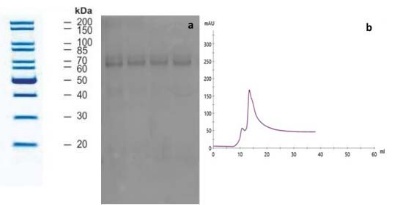
**

Fig S3.(a) Purified fractions of p62 run on a 12.5% SDS-PAGE gel (b) Elution profile of p62 after size exclusion chromatography
